# Oral Health Factors Related to Rapid Oral Health Deterioration among Older Adults: A Narrative Review

**DOI:** 10.3390/jcm12093202

**Published:** 2023-04-29

**Authors:** Jhanvi P. Desai, Rohit U. Nair

**Affiliations:** Department of Preventive and Community Dentistry, The University of Iowa College of Dentistry and Dental Clinics, Iowa City, IA 52242, USA

**Keywords:** oral hygiene, root caries, tooth loss, xerostomia, dependent oral care, ill-fitting partial dentures, dental care utilization

## Abstract

Older adults who face systemic health issues and lack adequate social support are at risk for oral health deterioration. How rapidly such changes take place depends on the severity of their medical condition and their ability to access oral health services in a timely manner. The management of dental caries and periodontal disease in this cohort is made complex by the interaction of local and host factors such as the presence of dry mouth, involvement of root surfaces, and altered wound healing. in addition to enhanced maintenance needs to avoid recurrence or progression. Tooth replacement can be beneficial in restoring oral function, allowing patients to enjoy a healthy and nutritious diet but requires careful consideration to avoid further damage to remaining dental units. Establishing a dental home for the older adult can facilitate routine surveillance, disease prevention, and patient/caregiver education to achieve oral health goals commensurate with overall health. This narrative review details oral health factors that are related to rapid oral health deterioration among older adults.

## 1. Introduction

Older adults constitute a unique and diverse demographic group in countries and societies around the world. Medical advances, financial and social support, and improvements in living conditions have all contributed to increased longevity, with global life expectancy increasing to nearly 73 years as of 2019 and that in OECD countries exceeding 79 years around the same time [1]. The evolution of medical and dental specialties that focus on patient-centered care for the older adult highlight the complexity of care needs that might be seen in this patient population [2].

**Figure 1:**
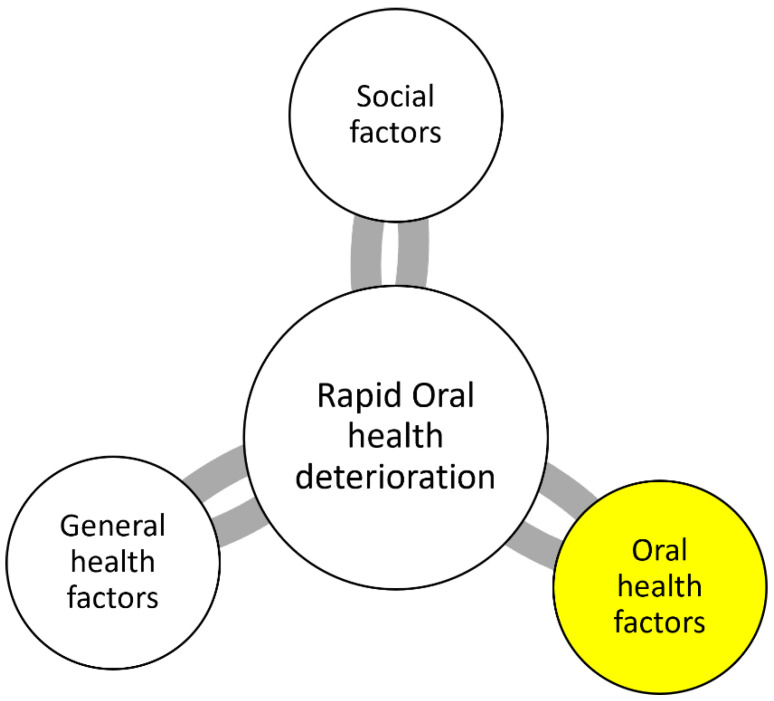
Factors contributing to rapid oral health deterioration. Adapted from Marchini et al [3].

**Figure 2:**
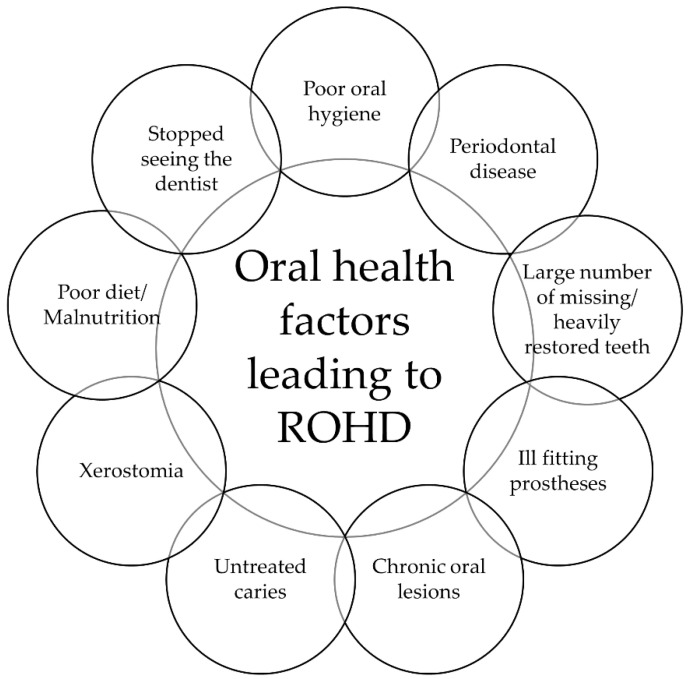
Oral health factors leading to rapid oral health deterioration. Adapted from Marchini et al [3].

The concept of rapid oral health deterioration (ROHD) provides a critical thinking framework for dental practitioners to systematically analyze the risk of older adult patients to experience progressive oral health decline that may be precipitated by a combination of systemic health events and oral health factors [3]. By leading the provider through an expert’s clinical decision-making style and weighing the relative impact of general health conditions, social support, and oral conditions (Figure 1), the ROHD model allows for risk stratification and facilitates broad guidance for treatment approaches while reconciling providers’ biases relating to patient care [4]. Increasingly, we see this and other similar models being successfully adopted in dental school curricula in response to the growing need for oral health services for older adults and other patients with special needs [5,6,7,8]. This review paper will focus on oral health factors related to ROHD among older adults as proposed by Marchini and colleagues, namely, oral hygiene, periodontal conditions, number of teeth/restorations, prosthetic status, presence of oral lesions, and utilization of dental services (Figure 2) [3].

## 2. Methods

Although there is general agreement over the fact that older adults experience oral health decline due to oral, systemic, and social factors, such changes are not documented in the literature exclusively using the term “rapid oral health deterioration”. To find information for this narrative review, we conducted a literature search within the PubMed database using search terms relating to each of the nine oral health risk factors originally proposed by Marchini et al. [3] in combination with terms that represent the older adult population. The search was limited to peer-reviewed publications that were indexed as articles or reviews. Terms used included “Dental Caries” [Mesh], “Dental Devices, Home Care” [Mesh], “Mouth Diseases” [Mesh], “No-Show Patients” [Mesh], “Oral Hygiene” [Mesh], “Periodontal Diseases” [Mesh], “Root Caries” [Mesh], “Toothbrushing” [Mesh] AND “Aged” [Mesh], “Aged, 80 and over” [Mesh], “Dental Care for Aged” [Mesh], “Frail Elderly” [Mesh], ROHD [tw], and rapid oral health deterioration [tw]. Abstracts of publications that resulted from this search were reviewed and shortlisted for inclusion based on the scope of this narrative review.

## 3. Discussion

### 3.1. Oral Health Factors related to ROHD among Older Adults

#### 3.1.1. Oral Hygiene

The likelihood of older adults maintaining a healthy, functional dentition throughout their lifespan depends on their ability to perform or receive daily oral hygiene care and to periodically access professional maintenance services [9]. This assumes particular significance for patients who have extensively restored dentitions or long-standing periodontal disease and those who are dependent for care. Oral health risk factors resulting from poor oral hygiene may predispose individuals to developing certain systemic health conditions, for example, diabetes mellitus or cardiovascular disease. Older adults can progress through the stages of rapid oral health deterioration after experiencing a major health event that diminishes their functional ability, requires new living arrangements/additional caregiver support, or disrupts normal oral physiology such as salivary flow. Although a causal relationship is difficult to establish, periodontal disease is a known risk factor for developing aspiration pneumonia, especially in institutionalized older adults with dysphagia. Regular oral hygiene maintenance can reduce the incidence of respiratory complications in this cohort [10]. 

Oral hygiene has been associated with cognitive conditions such as Alzheimer’s disease (AD) that affect the patient’s ability to perform activities of daily living [11]. The chronic inflammatory potential of periodontal disease may be somewhat associated with the risk of developing such progressive neurocognitive disorders, and poor oral health has been frequently cited as a modifiable risk factor for developing dementia [12,13,14,15,16]. Although much can be achieved through preventive approaches, the task of ensuring routine oral hygiene maintenance in older adults with cognitive disorders poses additional challenges. A team approach that involves the patient, caregivers, dental professionals, and occupational therapists may be beneficial when trying to restore oral self-care routines in such patients but might need to be modified based on the severity of their cognitive condition [17,18].

In the case of frail and/or dependent older adults, providing oral hygiene training to nursing assistants at long-term care facilities has been shown to improve residents’ oral health status [19]. Caregivers’ own attitudes toward dependent individuals’ oral health have been linked to their own oral health behaviors [20]. Poor or worsening manual dexterity can be the result of experiencing stroke, progressive musculoskeletal conditions like rheumatoid or osteo arthritis, neurological conditions like Parkinson’s disease, chronic pain such as that from fibromyalgia, etc. This can hinder older adults’ ability to independently perform daily oral hygiene and may necessitate the use of suitable oral hygiene aids [21]. It has been suggested that this cohort’s caries risk increases as they become functionally dependent toward the end of life [22]. Oral hygiene aids with physical modifications, such as adaptive toothbrush handles, triple-headed toothbrushes, Collis-curve brushes, floss holders/single-use floss picks, interdental brushes of varying sizes, water flossers, and electric toothbrushes, all facilitate better plaque removal in these individuals. Chemotherapeutic aids such as 1.1% sodium fluoride dentifrices (5000 ppm) and 0.12% chlorhexidine gluconate mouth rinse are known to be effective in providing additional protection against caries progression and periodontal disease. Since these are prescription-based agents, the patient and caregiver should be advised on the appropriate use of these products by the provider. Moreover, the dental provider will need to actively monitor for any side effects associated with these products [23,24].

A well-established oral hygiene routine that consists of brushing with a fluoridated toothpaste and flossing, either independently or with assistance from a caregiver, can deter the immediate impact of systemic health events on oral health until the patient’s primary dental care provider can assess their new needs and modify existing approaches to care [25].

#### 3.1.2. Periodontal Disease

Periodontal disease is one of the direct consequences of poor oral hygiene. A survey of US adults aged 65 years and over found that more than 60% of this group experienced some form of periodontal disease of varying severity [26]. While this may be the result of years of unmet oral hygiene needs, it poses a special risk for oral health decline in this population. Guarding the aging dentition from periodontal disease is particularly challenging considering the impact that aging has on wound-healing mechanisms, bone physiology, the oral microbiome, xerostomia, and the ability to ensure plaque control [27].

From an oral health standpoint, in the absence of adequate hygiene maintenance, local and systemic factors result in progressive deterioration of the supporting dental tissues causing gingival recession, bone loss, and increased tooth mobility. The functional impact of these changes is seen in older adults’ ability to chew food adequately and a tendency to choose softer foods that are easier to masticate but which may be rich in fermentable carbohydrates. The nutritional value of these food choices aside, there is an increased risk for root caries in the periodontally compromised dentition especially if accompanied by dry mouth [28].

Untreated periodontal disease can lead to tooth loss. Although it is possible to function on a reduced dentition, the presence of well-distributed, opposing dental units offers patients a better chance of sustaining a favorable oral health-related quality of life. The ability to function with a removable partial prosthesis also depends on the health of abutment teeth. Periodontal disease around strategic teeth can result in tooth mobility or gradual shifting under traumatic occlusal forces. Long-term changes in tooth position due to periodontal disease may render removable prostheses unusable, further affecting older adults’ quality of life. The presence of a removable prosthesis has also been shown to negatively impact the periodontal condition of abutment teeth, resulting in greater gingival recession, clinical attachment loss, caries, and fractures as compared to non-abutment teeth. A degree of manual dexterity is required to independently place and remove mechanically retained prostheses, to clean dentures, and to perform oral/peri-implant hygiene procedures. It is necessary to factor in patient’s ability to maintain oral hygiene, their adherence to a recall schedule, and the need for repairs and other maintenance procedures for the prosthesis when determining the periodontal prognosis of the abutment teeth [29].

From a systemic health perspective, active management of periodontal disease by performing periodic in-office scaling, root planning, and maintenance procedures can improve glycemic control, at least in the short-term, by minimizing a source of chronic inflammation [30,31]. A consensus report by the International Diabetes Federation and the European Federation of Periodontology confirms the safety and efficacy of periodontal therapy in diabetic patients and attests to modest improvements in HbA1C values up to 3 months after therapy [32]. Chaffee et al. studied the association between chronic periodontal disease and obesity and confirmed a positive relation between the two although it is difficult to infer if either condition poses a risk for the other [33].

#### 3.1.3. Presence of Untreated Dental Caries

Risk factors such as high sugar consumption, reduced salivary flow, reduced oral clearance due to lack of oral hygiene maintenance and/or xerostomia, reduced salivary buffering capacity, use of removable of prosthesis, and lack of fluoride exposure all contribute to both coronal and root caries development in older adults [34]. In fact, higher coronal caries experience in older adults is considered a risk predictor for root caries [35]. The combination of these risk factors can accelerate oral health deterioration.

Data from the National Health and Nutritional Examination Survey (NHANES) 2011–2016 highlighted the wide prevalence of dental caries among older adults; 96% of this cohort had experienced dental caries, and nearly one in every six individuals had untreated dental caries [36]. A sugar-rich diet allows demineralization of teeth to outpace remineralization resulting in caries. Thus, a carbohydrate-rich diet which may be softer in consistency and easier to prepare, may be preferred by patients with functional disability that results in challenges in mastication. However, this translates into more sugar intake which can magnify the risk of caries. In addition, other risk factors associated with this disease in older adults include belonging to disadvantaged sociodemographic groups, presence of systemic diseases, and lack of general and preventive oral health behaviors [37,38].

Adults over 65 years of age also experience the highest burden of untreated root caries among all age groups [39]. Root caries has a multifactorial etiology and is subject to some unique dental anatomical, histological, and microbiological features. Some habits, especially the use of tobacco in older adults, has been reported to have a positive correlation with root caries. Oral hygiene practices such as daily toothbrushing and interdental cleaning are linked with a lower incidence of root caries. Fundamentally, root caries develops on teeth that have had clinical attachment loss, a mechanism that is different from that related to other smooth surface caries. The presence of periodontal disease with biofilm formation and retention, as well as a lack of routine in-office cleaning, has a positive correlation to root caries experience [40]. Active root caries lesions require prompt intervention owing to their potential for rapid extension and involvement of nearby pulpal and periodontal tissues. The incidence of root caries can be reduced by modifying patients’ dietary habits, promoting self-care, and utilizing preventive services. Even without tooth loss, the presence of untreated caries increases the risk of experiencing pain and swelling and has the potential to affect an older individual’s quality of life.

#### 3.1.4. Restorative Status

Oral health-related quality of life is positively impacted by the retention of healthy natural teeth [41]. Depending on the extent of tooth loss, older adults may be rehabilitated with conventional or implant-supported complete/partial dentures, tooth or implant-supported fixed dental prostheses, or single-unit restorations. Complete denture prostheses are a comprehensive solution that minimize the disease burden resulting from poorly maintained dental hard tissues. These are, however, limited by the quality and stability of the foundational tissues they are designed to function on. Chronic changes in the supporting tissues because of residual ridge resorption makes it necessary for extensive removable denture prostheses to be periodically maintained with occlusal adjustments, relines, etc. The loss of retention encountered with removable prostheses can be overcome, to some extent, using tooth overdenture abutments with pre-machined attachments. This approach, however, requires careful maintenance to avoid developing recurrent caries/periodontal disease in the abutment teeth and subsequent prosthetic failure.

For the edentulous patient, implant supported overdentures offer a reliable way to improve retention and stability by placing multiple fixtures and compatible retentive elements along the span of the prosthesis [42]. These too can fail if not adequately maintained. Implant therapy itself may not be a feasible option for frail older adults due to the complexity of procedures involved, associated comorbidity, and increased need for peri-implant and prosthetic maintenance. Careful case selection, cleansable prosthetic design, personal and professional care regimens, and patient education will be critical to ensuring that middle-aged individuals who now have implant-supported restorations are not at risk of developing peri-implant disease or prosthetic failure as they approach old age and frailty themselves. Boven et al. report that although implant-supported dentures vastly improved patient comfort, improved patient satisfaction did not always improve their general or health-related quality of life, suggesting that there are other factors at play [43].

The shortened dental arch concept can be an effective treatment approach for the partially dentate patient who is unable to receive fixed prostheses and is maladaptive to removable prostheses [44]. Studies have shown that dental arches comprising the anterior and premolar regions meet the requirements of a functional dentition [45]. Research on occlusal stability, masticatory efficiency, and temporomandibular joint function support the design and maintenance of shortened arches in patients receiving fixed prostheses, providing an alternative to distal extension RPDs with which patients tend to be non-compliant. Moynihan et al. systematically reviewed the impact of wearing dentures on dietary intake but could not discern a clear association between the two. Nevertheless, the presence of stable, retentive tooth replacements can improve the overall experience of eating and restore esthetics where desired [46].

#### 3.1.5. Functional Dentition and Nutritional Status

Although there has been a significant global decline in the prevalence and incidence of severe tooth loss, older adults continue to be the most likely group to experience such adverse oral health events [47]. While this might be the outcome of accumulated oral health needs across their lifespan, the consequences of tooth loss have a significant impact on older adults’ quality of life at a time when they may not be able to adapt to rehabilitative procedures owing to other systemic/functional limitations. The presence and severity of tooth loss have also been associated with mortality resulting from metabolic, digestive, and cardiovascular disease [48,49].

The relationship between nutrition and oral health is bidirectional and dynamic in nature. A balanced diet helps preserve oral health while a functioning oral cavity enables the gastrointestinal system to assimilate dietary elements resulting in benefits to one’s overall health. While oral health, diet, and nutrition are closely linked, food choices vary widely among people due to personal preferences, cultural influences, access to food resources, and several other factors [50].

Marcenes et al. studied the relationship between dental status, food selection, nutrition, and body mass index (BMI) in a national sample of adults aged 65 years and over living in Great Britain. They found that older adults with a functional dentition comprising 21 or more teeth were likely to have a healthier diet that was rich in fruits and vegetables and have a favorable BMI [51]. While it is intuitive to link the number of remaining functional dental units with older adults’ risk of developing malnutrition, the multifactorial nature of the latter condition must be emphasized. Bakker et al. found in a cross-sectional observational study of 1325 community-dwelling adults aged 75 years and over that although malnourished older adults reported problems with speech and mastication, there was no significant association between malnutrition and oral health problems, including edentulism. However, respondents’ health-related quality of life (HRQoL) was associated with malnutrition, suggesting that a combination of oral, systemic, and social factors was likely at play [52].

Gil-Montoya et al., in a cross-sectional study of 2860 adults in Spain aged 65 years and older, validated the use of the oral health-related quality of life (OHRQoL) assessment to identify older adults who are at risk of malnutrition due to oral problems [53]. In addition to the number of remaining teeth, other factors, such as presence of xerostomia, poor appetite or trouble swallowing, also play an important role in shaping older adult’s likelihood of facing nutritional problems [54,55].

Impaired dental status among older adults is linked to chewing difficulty, dysphagia, and oral pain due to chronic inflammation and persistent infections, all of which lead to dietary limitations. Masticatory efficiency is affected by tooth loss, number of functional occluding teeth, and presence of a prosthesis. With limited masticatory efficiency, older adults may be at risk of making poor dietary choices. Softer foods often tend to be high in sugars and fats and low in protein and fiber. This in turn affects systemic health by increasing risk of coronary artery disease, diabetes, gastrointestinal issues, etc. [56]. In a nationwide survey of 4820 adults aged 50 years and over who had at least 18 teeth or who wore dentures, Sahyoun et al. found that self-perceived ill-fitting of dentures negatively impacted this cohort’s inclusion of vegetables and other essential nutrients in their diet, resulting in significantly lower serum levels of vitamins C and E, beta carotene, folic acid, etc., in comparison to dentate peers [57].

In a study conducted by Maitre et al. in France, 23% of older adults in the study sample were picky eaters with food selectivity being highest among nursing home residents. The findings of this study reiterated that greater food selectivity correlates with an increase of malnutrition risk and is parallel to the effect of eating difficulties on malnutrition [58]. Sheiham et al. in Great Britain showed that older adults who had less than 21 natural teeth were on an average 3 or more times likely to be obese while individuals with fewer than 11 teeth were significantly more likely to be underweight [59].

Older adults face a higher risk for dehydration due to reduced muscle mass, decline in kidney function, disability, and reduced thirst [60]. This, in combination with factors including antisialagogue drugs, radiation therapy, or comorbidities such as Sjogren’s syndrome, can predispose older adults to xerostomia. Having a poor dentition may result in body image issues, decreasing social interactions, and being at risk of psychosocial disorders. Conditions such as loneliness and depression may cause affected individuals to resort to non-nutritional binging on comfort food, resulting in a vicious cycle that further compromises oral and general health [61].

#### 3.1.6. Conditions Affecting Oral Soft Tissues

Candidiasis is a very common mycotic infection of the oral cavity [62]. Although it can be seen across the lifespan, there are several risk factors that predispose older adults developing oral candidiasis in [63]. These include long-term antibiotic therapy, poor oral/denture hygiene, dry mouth, chronic immunosuppressive conditions, and diabetes mellitus, among others, all of which are commonly encountered in this cohort. Buranarom et al., in a cross-sectional study of 53 independent adults aged 65 years and over, reported that patients with hyposalivation were nearly four times more likely to have oral Candida colonization [64]. A similar trend was noted in a cohort study by Deng et al., where more than half the number of subjects who received irradiation to the oral cavity developed oral candidiasis compared to only about 12% of the nonirradiated group. Xerostomia, mouth and throat soreness, and dysphagia were significantly higher in the former group, underscoring a complex situation that favors the progression of such infections in the medically compromised host [65]. Effective management of oral Candida infections can also have a beneficial effect on salivary flow. Ohga et al. reported significantly higher whole salivary flow rates following antifungal therapy with miconazole gel to eliminate oral Candida spp. in a group of 52 mostly older adult patients. This was accompanied by improvement in self-reported xerostomia and oral pain scores [66].

Denture stomatitis is an inflammatory, erythematous condition of denture-bearing oral mucosal tissues. As with candidiasis, it too has a complex etiology and could be the result of denture materials, poorly fitting dentures, inadequate denture hygiene, continuous wearing of removable dentures, poor denture plaque control, or bacterial and fungal contamination of denture surfaces [67]. All these factors facilitate the growth of opportunistic pathogens such as Candida albicans on affected tissues. The combination of mucosal infections and prosthetic malfunction makes this a significant quality of life issue for older denture wearers, especially when declining systemic health impacts their ability to receive oral health services.

Angular cheilitis is an inflammatory condition affecting the commissures of the mouth. It can result from a deficiency of essential nutrients including iron and vitamin B12, drooling, and denture-related issues, among other reasons. In denture wearers, the lack of adequate vertical dimension at occlusion can result in inversion of the angles of the mouth causing maceration and erythema. Chronic overloading of maxillary anterior denture-bearing tissues leads to the replacement of alveolar bone with fibrous tissues resulting in a “flabby ridge” that compromises denture stability [68]. Chronic irritation can result in the fibrous growths in vestibular areas adjacent to denture borders, causing discomfort and necessitating surgical removal prior to remaking or repairing prostheses. Unresolved mechanical tissue trauma, as with chemical irritants such as tobacco and alcohol, can result in precancerous leukoplakic and erythroplakic changes and subsequently increase the risk for oral squamous cell carcinoma [69]. In an analytical cross-sectional study of 821 participants aged 60–100 years in Brazil, Saintrain et al. showed that injuries to the oral cavity were significantly associated with age, retirement status, level of education, and the presence of dentures [70]. Furthermore, teeth with lost restorations, fractured teeth, or those with erosive or attritional wear can cause traumatic ulcers on the tongue/cheek mucosa if left unaddressed.

Older adults may need additional surveillance of soft tissue injuries to prevent progression and to monitor suspected malignant lesions to ensure timely referral for management. Older adults are particularly vulnerable to head and neck cancers with close to half of all new cases diagnosed in the United States between 1973 and 2008 occurring in patients aged 65 years and over [71]. This number is likely to rise as the global aging population increases and more sophisticated diagnostic tools become available. Considering the impact that multimodality therapy–surgery, radiation, chemotherapy–can have on oral soft tissues, supportive care such as the management of mucositis, xerostomia, and dysphagia is critical to the patient’s quality of life [72]. As discussed earlier, all of these are also directly related to their risk for developing secondary oral complications such as dental caries and periodontal disease.

#### 3.1.7. Xerostomia

Xerostomia is the subjective perception of oral dryness that may or may not be accompanied by an actual decline in salivary flow. Ship and colleagues estimated at least 30% of older adults experience dry mouth, with this number being as high as 72% among institutionalized older adults. In the same study, the prevalence of xerostomia was 100% in older adults with Sjogren’s syndrome and those who had experienced cancer-related head and neck radiation [34,35]. Salivary gland hypofunction can lead to xerostomia; however, both are not necessarily correlated. It is important to note that both xerostomia and salivary hypofunction cause decrease in oral health-related quality of life, and the perception of dry mouth may not only be a result of salivary quantity but changes in the salivary composition as well.

Older adults are more likely to be prescribed multiple medications, as they may be managing concomitant systemic conditions. Xerostomia associated with polypharmacy is common in patients who use of five or more medications which most often have antisialagogic effects. Classes of drugs like anticholinergics, antidepressants, antipsychotics, diuretics, anxiolytics, antihistamines, antihypertensives, and analgesic drugs are frequently implicated in causing dry mouth in older adults. Since older adults are more likely to take multiple prescription medications as compared to the rest of the population, they are also most vulnerable to the side effects of these drugs and their impact on oral disease experience [73]. History of radiation to the head and neck, diseases of salivary glands, diabetes, alcoholic cirrhosis, and autoimmune disorders such as lupus and Sjogren’s syndrome are some biological factors leading to dry mouth. Certain social and psychological factors, such as depression, stress, and anxiety, are also recognized as etiologic factors [74].

Reduced salivary flow allows micro-organisms to aggregate and adhere better in the oral environment, which causes the biofilm to stagnate longer, leading to loss of tooth structure, gingivitis, periodontal disease, dental caries, and halitosis. Since immunological functions of saliva are altered due to age as well as salivary hypofunction, older adults face a higher risk for opportunistic infections such as candidiasis because of hyposalivation. Candida species often harbor in the fissures of the tongue, leading to symptoms of burning tongue [75]. Studies highlight dry mouth as a local contributory factor in debilitating oral conditions such as burning mouth syndrome, which is characterized by the presence of a burning sensation of the oral mucosa in the absence of clinically apparent mucosal alterations. This disorder is often a challenge to diagnose and negatively impacts the patient’s oral health-related quality of life [76]. In edentulous patients, reduced quality and quantity of saliva can negatively impact denture retention and cause discomfort while chewing due to insufficient lubrication between the denture base and supporting tissues. Depending on the severity of the condition, older adults who experience xerostomia may alter food choices, preferring items with higher moisture content to be able to comfortably masticate food and experience a variety of flavors and textures [77].

#### 3.1.8. Utilization of Dental Services

As the global older adult population grows and continues to retain teeth for longer, their oral health needs are projected to increase as well [78]. However, a disparity exists when it comes to the utilization of dental services by older adults. There are many factors that affect older patients’ ability to see a dentist or establish a dental home. As per the Health Policy Institute’s 2019 Annual Dental Industry report, the utilization of dental services among older adults was influenced by dental insurance, household income, perceived affordability, and overall health outcomes. Older adults on public dental insurance programs utilized dental care the least, and race and/or ethnicity-related disparities in dental service utilization were apparent [79,80,81,82].

According to the CDC, there is a higher prevalence of untreated caries, edentulism, and periodontal disease among older adults from minority groups [83]. There is some association between poor oral health, self-perceptions of oral health, and affordability among older adults coming from disadvantaged ethnic minorities. This is a barrier to oral care by itself since such patients may not seek out oral care unless they are facing a dental emergency [84]. Occupational status and education among older adults were also found to be important predictors of the utilization of dental services [85]. Rural-dwelling older adults as well as those living in long-term care facilities typically were reported to have fewer visits to the dentist, making location of residence a predictor in understanding dental use by this group. Residents of long-term care facilities were found to face a combination of other barriers, such as poor functional status, difficulty in making visits to dental providers, and the unavailability of dentists who provide dental care within the nursing home settings [86].

From a functional standpoint, poor systemic health and multiple comorbidities are predictors of low dental service uptake among older adults. A study by Kuthy et al. suggested that individuals who had frequent medical visits and spent more resources on medications/medical visits tended to use dental services less. This can be correlated to the shift in focus from oral health to chronic conditions that impair activities of daily living [87]. One psychosocial predictor of using dental services among older adults is a social support network. Older adults who experience social isolation due to loss of a spouse, close friends, or relocation to a new community tend to utilize dental services less. This could be due to a lack of interpersonal ties and social connectedness resulting in lapsed oral health behaviors including seeing a dentist on a regular basis [88].

Lastly, dental providers’ attitude can be both an enabler and barrier to the use of dental services among older adults. Dentists’ beliefs, stereotypes, and skill levels with older patients can encourage or discourage use of dental services among this population. This is also compounded by shortage of skilled geriatric oral health care professionals making it harder for patients to access those providers with expertise in the geriatric dentistry [89].

## 4. Summary

Oral health and systemic health are closely related. When viewed through the lens of the rapid oral health deterioration conceptual framework, it becomes possible to identify specific risk factors that increase older adults’ likelihood of experiencing a sharp decline in their oral health following a significant health event (Table 1). Good oral hygiene, performed independently or with help from a caregiver, offers protection from dental caries and periodontal disease. Retaining healthy teeth and replacing missing teeth strategically can facilitate speech, function, and esthetics while improving older adults’ quality of life. General health conditions or therapies to manage these can alter normal oral physiology and render older adults susceptible to unwanted side effects such as xerostomia. A combination of these and other social factors plays a role in their ability to choose and consume a nutritious diet. Oral soft tissue lesions require prompt management not only to ensure comfortable functioning with removable prostheses but also to minimize the risk of developing cancer from long-standing irritation. It is essential that older adults have a dental home in addition to a medical home to ensure that they are receiving timely preventive oral care and have access to the expertise needed to arrest rapid oral health deterioration.

## Figures and Tables

**Table 1:** Interrelated nature of oral factors contributing to rapid oral health deterioration.

Oral Health Factors Leading to ROHD	Contributing Factors	Effects on Oral Health
**Poor oral hygiene**	Major health event, frailty, decreased manual dexterity, dependence on daily oral care, cognitive decline	Plaque retention, gingival inflammation, risk for periodontal disease, increased caries risk, halitosis
**Periodontal disease**	Inadequate oral hygiene maintenance, systemic risk factors or chronic inflammatory conditions, metabolic factors such as obesity, diabetes mellitus	Occlusal instability, tooth movement, tooth mobility, tooth loss, halitosis
**Number of remaining teeth/ restorations**	Untreated dental caries, untreated periodontal disease, access to dental services	Multisurface restorations needing maintenance, interrupted dental arches, reduced masticatory ability, psychological impact of tooth loss
**Sequelae of wearing fixed or removable dental prostheses**	Condition of denture-bearing tissues, patient perception, ill-fitting dentures, worn-out artificial teeth, difficulty with placing and removing dentures, poor denture hygiene	Maladaptation to dentures, failure of abutment teeth, denture stomatitis, reduced masticatory ability, oral candidiasis
**Presence of oral lesions**	Poor denture hygiene, denture irritation, loss of occlusal vertical dimension, continuous denture usage, head and neck cancer, radiotherapy to this region	Candidiasis, traumatic ulcers, angular cheilitis, flabby tissue, dry mouth, burning mouth syndrome, radiation mucositis, decreased OHRQoL
**Untreated dental caries**	Poor oral hygiene maintenance, untreated periodontal disease, high sugar exposure, dry mouth, presence of prostheses, low access to dental care	Pain, reversible or irreversible pulpitis, increased risk for tooth fracture, tooth loss
**Xerostomia**	Polypharmacy, xerogenic medications, head and neck radiotherapy, salivary gland diseases, diabetes, autoimmune disorders such as Sjogren’s syndrome or lupus	Dental caries, difficulty wearing dentures, decreased OHRQoL
**Dietary choices**	Loss of functional occluding units, dietary choices which have high sugar, insufficient calorie intake, availability of caregiver support	Increased caries risk, obesity, nutritional deficiencies related to proteins, vitamins, and minerals; dehydration; increased risk for malnutrition; frailty
**Inability to utilize dental services**	Caregiver availability, financial limitations, transportation barriers, institutionalization, poor systemic health, dental workforce issues	Discontinued dental visits, lack of surveillance of progressive oral conditions

## Data Availability

Not applicable.
